# Mean Intracranial Volume of Brain among Patients with Normal Magnetic Resonance Imaging Referred to the Department of Radiology and Imaging of a Tertiary Care Centre

**DOI:** 10.31729/jnma.8357

**Published:** 2023-12-31

**Authors:** Om Biju Panta, Bibek Gurung, Shahjan Raj Giri, Abhishek Adhikari, Ram Kumar Ghimire

**Affiliations:** 1 Department of Radiology and Imaging, Nepal Mediciti Hospital, Bhaisepati, Lalitpur, Nepal

**Keywords:** *brainstem*, *cerebellum*, *cerebrum*, *magnetic resonance imaging*

## Abstract

**Introduction:**

The measurement of brain volume is an important aspect of the assessment of brain structure and function. However, limited data is available on brain volumetry in the Nepalese population. The study aimed to find the mean intracranial volume of the brain among patients with normal magnetic resonance imaging referred to the Department of Radiology and Imaging of a tertiary care centre.

**Methods:**

A descriptive cross-sectional study was conducted among patients with normal magnetic resonance imaging referred to the Department of Radiology and Imaging in a tertiary care centre. All magnetic resonance imaging of the brain during the study period was reviewed by a radiologist. Magnetic resonance imaging with abnormal findings, clinical signs of neurological deficit, dementia and psychiatric symptoms were excluded from the study. A convenience sampling method was used. The point estimate was calculated at a 95% Confidence Interval.

**Results:**

Among 285 Magnetic Resonance Imaging datasets, the mean intracranial volume was 1286.30±129.88 cc (1271.22-1301.38, 95% of Confidence Interval). The mean cerebral volume was 985.06±106.4 cc, cerebellar volume was 126.99±13.05 cc and brain stem volume was 19.97±2.54 cc.

**Conclusions:**

The mean intracranial volume of the brain among patients with normal magnetic resonance imaging was found to be lower than other studies done in similar settings.

## INTRODUCTION

The brain's size, structure, and composition play pivotal roles in health and disease.^1^ Volumetric measurements are important, especially in diseases like schizophrenia and dementia.^2,3^ Accurate measurement of different brain structures is essential for studying the underlying mechanisms of neurological disorders and for monitoring the progression of such diseases.^3^ Magnetic Resonance Imaging (MRI) stands as a pivotal tool in the diagnosis and assessment of various neurological conditions, providing detailed insights into the structural integrity of the brain.

Exploration of the size and structure of the brain among individuals with unremarkable MRI scans can offer invaluable insights. There are limited studies related to the intracranial volume of the brain to date.

The study aimed to find the mean intracranial volume and volume of the brain among patients with normal magnetic resonance imaging referred to the Department of Radiology and Imaging of a tertiary care centre.

## METHODS

A descriptive cross-sectional study was performed on patients with magnetic resonance imaging (MRI) referred to the Department of Radiology and Imaging of a tertiary care centre from 1 January 2019 to 31 December 2020. MRI scans performed for headache; and facial pain were included in the study. MRI scans with abnormalities, with clinical presentation of dementia, psychological disorder, epilepsy and focal neurological deficit were excluded from the study and patient with a history of severe head injury, and epilepsy under medication was identified in the clinical case sheet and were excluded from the study. A convenience sampling method was used. The sample size was calculated using the following formula:


$$\begin{array}{ll} \text{n} & ={\text{Z}}^{2}\times \frac{\sigma }{{\text{e}}^{\text{2}}}\\ & =1.96^{2}\times \frac{173^{2}}{25^{2}}\\ & =184 \end{array}$$

Where,

n = minimum required sample size
Z = 1.96 at 95% Confidence Interval (CI)
σ = standard deviation taken from previous study^4^
e = margin of error

The calculated sample size was 184. However, we have included 285 MRI datasets in this study.

Clinical indication for MRI recorded in the radiological requisition and clinical case sheet of the patients in hospital records from pre-anaesthetic care was reviewed. All MRI scans were performed using the Siemens Skyra 3T MRI machine. The sequence used for volumetric assessment was a T1-weighted 3D MP-RAGE with the following parameters: TR= 2300 msec, inversion time= 900 msec, flip angle= 8°, bandwidth = 200 Hz/pixel, FOV= 240×240 mm, matrix= 256×256, resulting in voxel dimensions of 0.9×0.9×0.9 mm, and an acquisition time of 4 min 44 sec. MRI data processing and volumetric analyses were performed using volBrain version 1.0.

The collected data were entered into Microsoft Excel 2017 and analysed using IBM SPSS Statistics version 26.0. The point estimate was calculated at a 95% CI.

## RESULTS

The mean intracranial volume of 285 patients with normal magnetic resonance imaging was 1286.30±129.88 cc (1271.22-1301.38, 95% CI). The mean age of the population was 39.52±14.09 years. Out of 285 patients, 190 (66.67%) were female while 95 (33.33%) were males. The mean age of males was 38.49±15.18 years and that of females was 39.95±13.41 years. The mean total brain volume in males was 1205.41±125.64 cc and in females was 1089.55±101.50 cc (Table 1).

**Table 1:** Brain volumetry (n= 285).

Parameters	Male mean±SD (cc)	Female mean±SD (cc)	Total mean±SD (cc)
Intracranial volume	1374.65±130.71	1242.13±104.72	1286.30±129.88
Total brain volume	1205.41±125.64	1089.55±101.50	1128.17±122.78
Grey matter	772.58±97.39	696.94±82.81	406.01±61.06
White matter	432.82±62.66	392.61±55.75	722.15±94.76
CSF	169.24±50.96	152.57±51.17	158.13±51.61
Cerebral volume	1055.81±104.88	949.68±88.12	985.06±106.4
Cerebrum right lobe	523.6±52.8	471.95±44.67	489.17±53.34
Cerebrum left lobe	532.21±55.36	477.73±44.52	495.89±54.73
Cerebellum	134.02±11.91	123.47±12.16	126.99±13.05
Cerebellum right lobe	66.17±6.54	60.9±6.21	62.66±6.56
Cerebellum left lobe	67.86±6.54	62.57±6.34	64.33±6.86
Brainstem	21.37±2.33	19.27±2.36	19.97±2.54

Intracranial volume among age group of <20 years is 1288.75±94.33 cc (Table 2).

**Table 2:** Age-wise distribution of brain volumetry (n= 285).

Brain morphometry	<20 years (19)	20-30 years (62)	30-40 years (78)	40-50 years (63)	50-60 years (38)	>60 years (25)
	mean±SD	mean±SD	mean±SD	mean±SD	mean±SD	mean±SD
Intracranial volume (cc)	1288.75±94.33	1289.71±126.71	1296.06±133.76	1303.43±136.41	1250.84±135.19	1268.73±123.55
Total brain volume (cc)	1172.74±80.24	1146.12±123.13	1137.78±125.61	1138.1± 115.09	1083.12±142.51	1063.24±91.07
Grey matter (cc)	776.59±61.47	734.62±93.53	726.04±98.51	725.99±83.67	679.66±117.13	692.66±64.89
White Matter (cc)	396.15±65.04	411.49±61.77	411.74±65.63	412.10±51.02	403.47±60.94	370.57±57.45
Cerebrospinal Fluid (cc)	116.02±29.18	143.59±54.58	154.28±48.97	165.33±38.43	167.72±46.64	205.49±61.94
Cerebral Volume (cc)	1029.70±85.62	997.19±111.25	991.15±112.55	993.57± 109.40	961.94±88.51	915.71±72.41
Cerebrum Right Lobe (cc)	509.81±43.94	493.90±56.15	492.73±57.02	492.56±54.40	477.98±43.54	459.09±40.06
Cerebrum Left Lobe (cc)	519.89±43.93	503.28±56.90	498.42±56.34	501.01±57.42	483.96±45.46	456.62±36.99
Cerebellum (cc)	132.33±11.33	126.90±13.48	129.21±12.61	126.72±14.42	124.80±10.57	120.25±11.98
Cerebellum Right Lobe (cc)	64.97±5.84	63.02±6.28	63.77±6.39	62.38±7.44	61.47±5.6	59.07±6.03
Cerebellum Left Lobe (cc)	67.36±5.59	63.88±8.09	65.44±6.38	64.33±7.19	63.33±5.29	61.18±6.12
Brainstem (cc)	19.48±2.11	19.67±2.74	20.33±2.58	20.08±2.45	20.22±2.61	19.32±2.34

## DISCUSSION

The mean intracranial brain volume in our study was 1286.30±129.88 cc which is lower than study reported in most previous studies.^5-8^ The mean total brain volume in our study was 1205.41±125.64 cc, while total brain volume in the Indian population in the previous study was 1442+/-143 cc.^6^ Similarly brain volume in the Chinese population was 1510 cc.^8^ This shows wide variation in brain volumes among various studies. This might actually be due to the variation in techniques that quantified the volume of the brain in different studies. One study in Europe has shown significant clinal variation in brain morphometry among Europeans but, there is limited data from Asia and particularly Nepal regarding craniometry and brain morphometry.^9^

The shape and size of the skull vary with race and ethnicity.^9,10^ A previous study comparing cranial volumes documented smaller intracranial volume in Asians compared to Westerners.^11^ The intracranial volume in this study was measured in Singapore and the United States. The total intra-cranial volume in that study in the young Singapore population was 1466.1 cc and in the United States population was 1594.84 cc. A study done in 2010 estimated the brain volume of 1136±121 cc, which is similar to our study.^12^ Grey matter volume in this study was 474.4±45.0 cc by surface-based representation measurement and 333.6±43.9 cc by volume-based representation which is lower than that found in our study. The difference in results is likely due to the use of different techniques. The automatic segmentation ("VolBrain" software) method used in our study is more advanced and more accurate than the manual technique used in the prior study. Another study done on anatomical specimens in 2013 estimated an average total cerebral hemispheric grey matter volume of 276.36 cc and white matter volume of 222.25 cc; which is comparable to the average hemispheric volume of cerebrum in our study.^13^

In a similar study carried out in children up to 18 years of age, the volume of the brain, grey matter, and white matter was comparable to our study, but the volume of the brain stem was higher in their study.^14^ These results suggest that despite the change in size of the skull, the brain volume is relatively constant in different races and ethnicities. The variation of brain volumetry among various races in the Nepalese population could be an interesting area of research.

The brain exhibits a high degree of variability in regional distribution and shape among individuals, with differences also seen among genders and ethnicities.^15,16^ The variation in shape and regional distribution has not been studied in our study and might be an area of future research to better understand brain structure.

The brain volume is known to progressively reduce with age. Previous studies also have shown a reduction in brain volume with ageing.^11^ Our study also found a decline in brain volume with age, particularly after the age of 60 years. The reduction of brain volume with ageing is an established fact, however, there are various factors that affect the rate of change and the biological age of the brain. Also, the volume of the brain at which cognitive decline begins is yet to be established. A larger prospective cohort study is needed to understand the age-related changes in the brain and factors associated with the rate of change.

There were several limitations to our study. Firstly, it was a hospital record-based study rather than a population-based study. Our hospital caters to mid to high-class urban patients, which might not be a true representation of the population. We did not actively interview the patients for clinical history and clinical indications were extracted from hospital records which might be a reason for bias. Also, we did not consider the nutritional status of the patients and family history of neurodegenerative disorders which might have an impact on the brain volume of the patient. Although our sample size was large enough, it was still insufficient to compare among ethnic and age groups.

## CONCLUSIONS

The mean intracranial volume of the brain among patients with normal magnetic resonance imaging was found to be lower than other studies done in similar settings.
